# CRISPR/Cas9 Screening for Identification of Genes Required for the Growth of Ovarian Clear Cell Carcinoma Cells

**DOI:** 10.3390/cimb44040108

**Published:** 2022-04-07

**Authors:** Ayako Kawabata, Tomoatsu Hayashi, Yoko Akasu-Nagayoshi, Ai Yamada, Naomi Shimizu, Naoko Yokota, Ryuichiro Nakato, Katsuhiko Shirahige, Aikou Okamoto, Tetsu Akiyama

**Affiliations:** 1 Laboratory of Molecular and Genetic Information, Institute for Quantitative Biosciences, The University of Tokyo, Tokyo 113-0032, Japan; ayakokawabata0702@jikei.ac.jp (A.K.); ykaks1128@gmail.com (Y.A.-N.); aiyamada@iqb.u-tokyo.ac.jp (A.Y.); shimizu-yasui@iqb.u-tokyo.ac.jp (N.S.); 2 Department of Obstetrics and Gynecology, Jikei University School of Medicine, Tokyo 105-8461, Japan; aikou@jikei.ac.jp; 3 Laboratory of Computational Genetics, Institute for Quantitative Biosciences, The University of Tokyo, Tokyo 113-0032, Japan; york@iqb.u-tokyo.ac.jp (N.Y.); rnakato@iqb.u-tokyo.ac.jp (R.N.); 4 Laboratory of Genome Structure and Function, Institute for Quantitative Biosciences, The University of Tokyo, Tokyo 113-0032, Japan; kshirahi@iqb.u-tokyo.ac.jp

**Keywords:** ovarian cancer, ovarian clear cell carcinoma, CRISPR/Cas9 system, proliferation, tumorigenesis

## Abstract

Epithelial ovarian cancer is classified into four major histological subtypes: serous, clear cell, endometrioid and mucinous. Ovarian clear cell carcinoma (OCCC) responds poorly to conventional chemotherapies and shows poor prognosis. Thus, there is a need to develop new drugs for the treatment of OCCC. In this study, we performed CRISPR/Cas9 screens against OCCC cell lines and identified candidate genes important for their proliferation. We found that quite different genes are required for the growth of ARID1A and PIK3CA mutant and wild-type OCCC cell lines, respectively. Furthermore, we found that the epigenetic regulator KDM2A and the translation regulator PAIP1 may play important roles in the growth of ARID1A and PIK3CA mutant, but not wild-type, OCCC cells. The results of our CRISPR/Cas9 screening may be useful in elucidating the molecular mechanism of OCCC tumorigenesis and in developing OCCC-targeted drugs.

## 1. Introduction

Epithelial ovarian cancer (EOC) is one of the most common gynecologic malignancies and has a poor prognosis [1,2,3,4]. It is classified into four major histological subtypes: serous, clear cell, endometrioid and mucinous [5]. Ovarian clear cell carcinoma (OCCC) shows particularly poor prognosis, and its 5-year survival rates for Stages III and IV are 20~30% [6,7,8,9,10]. OCCC responds poorly to conventional platinum-based therapies and is associated with endometriosis, paraneoplastic hypercalcemia and thromboembolism [6,7,8,9,10]. The incidence of OCCC is low in Western countries (~5%), but high in Asia, especially in Japan (~25%) [6,11,12].

Mutations in a number of oncogenes and tumor-suppressor genes are involved in the development of OCCC [6,13,14]. Mutational inactivation of the AT-rich interaction domain 1A (ARID1A) and activation of phosphatidylinositol-4,5-bisphosphate 3-kinase catalytic subunit α (PIK3CA) are most frequently found (about half of the cases of OCCC). ARID1A is a subunit of the SWI/SNF chromatin remodeling complex, which regulates gene expression by modulating the accessibility of promoters to transcription factors [15]. Thus, mutational inactivation of ARID1A results in dysregulation of gene expression. PIK3CA encodes the catalytic subunit p110α of phosphatidylinositol 3-kinase (PI3K). Gain-of-function mutations in PIK3CA enhance its PI3K activity and activates the downstream AKT pathway [16]. Mutations in ARID1A and PIK3CA frequently cooccur in OCCC and are considered to cooperate in tumorigenesis [6].

Based on the knowledge of the molecular mechanisms of OCCC tumorigenesis, research is being conducted to identify new molecular targets for therapeutic agents. For example, it has been shown that ARID1A-mutated OCCC cells are vulnerable to inhibition of the DNA repair proteins PARP1/2 and ATR, and the epigenetic factors EZH2, HDAC2, HDAC6 and BRD2 [17]. Additionally, it has been reported that EGLN1, which encodes prolyl hydroxylase domain-containing protein 2 (PHD2), plays an important role in the proliferation of a subset of OCCC in vitro, presumably by negatively regulating HIF1A [18]. It has also been reported that the majority of OCCC cells are sensitive to mTORC1/2 inhibition both in vitro and in vivo [19].

As a basis for elucidating the molecular mechanism of OCCC tumorigenesis and developing therapeutic agents, we set out to identify novel molecular targets critical for the proliferation of OCCC using the CRISPR/Cas9 system.

## 2. Materials and Methods

### 2.1. Cell Culture

TOV21G cells were cultured in 1:1 mixture of MCDB 105 medium and Medium 199 supplemented with 15% FCS. OVISE cells were cultured in Dulbecco’s modified Eagle’s medium (DMEM) supplemented with 10% FCS. JHOC5 cells were cultured in RPMI1640 medium supplemented with 10% FCS. ES2 cells were cultured in McCoy’s 5A medium supplemented with 10% FCS. These cell lines are widely used as OCCC cell lines [20], but there are several papers questioning the origin of ES2 [21,22].

### 2.2. Pooled Genome-Wide CRISPR/Cas9 Knockout Screen

**Figure 1:**
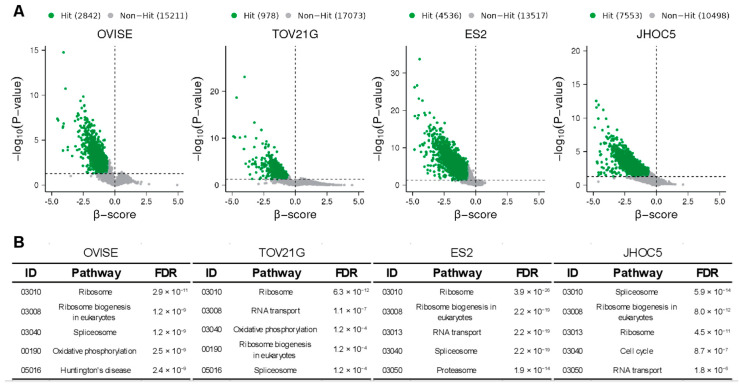
Genome-wide CRISPR/Cas9 screens using OCCC cell lines. (**A**) CRISPR-Cas9 screening results analyzed by MAGeCK-MLE. Green points represent significantly depleted genes (beta ≤ −0.6, *p*-value ≤ 0.05). Horizontal dotted lines represent *p* = 0.05. (**B**) Results of PATHWAY analysis of the top 1000 hit genes in (**A**) using STRING-db. The top 5 pathways are shown along with their associated false discovery rates (FDRs).

**Figure 2:**
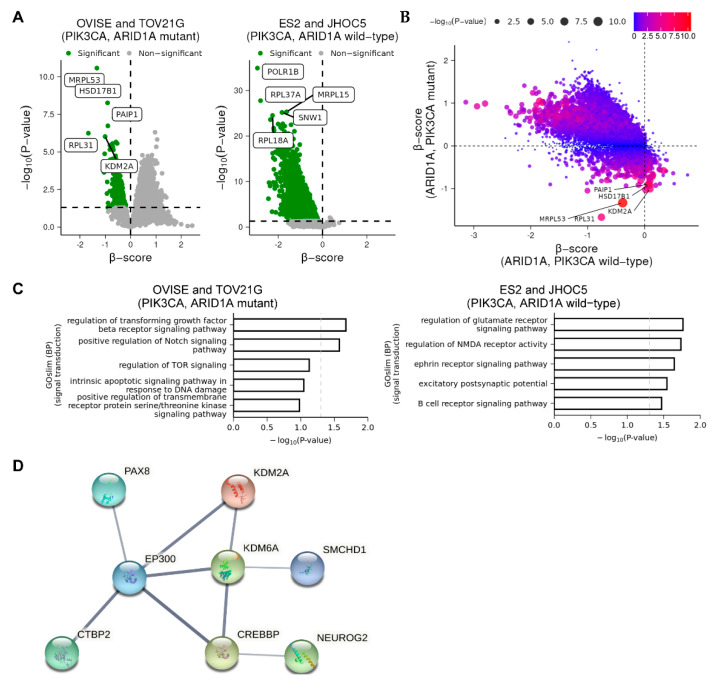
Effects of mutations in ARID1A and PIK3CA on CRISPR/Cas9 screening. (**A**) Results of CRISPR/Cas9 screens in OCCC cell lines with and without mutations in ARID1A and PIK3CA. Green points represent significantly depleted genes (β ≤ −0.6, *p*-value ≤ 0.05). Horizontal dotted lines represent *p* = 0.05. (**B**) Scatter plot analysis of the results shown in (**A**). (**C**) GO analysis of the results shown in (**A**,**B**). (**D**) STRING-db analysis of protein–protein networks containing KDM2A. The genes significantly depleted in ARID1A and PIK3CA mutant cells (β score ≥ 0) but not in ARID1A and PIK3CA wild-type cells (β score ≤ 0) were subjected to STRING-db analysis of protein–protein networks.

CRISPR-Cas9 screens in OCCC cell lines were performed using the human genome-wide CRISPR-Cas9 library TKOv3 (Addgene #90294) [23] according to a protocol deposited in Addgene website (https://media.addgene.org/cms/filer_public/71/a8/71a81179-7a62-4d75-9b53-236e6f6b7d4d/tkov3_guide_sequence.xlsx, accessed on 1 March 2022). Briefly, for each screen replicate, 2.0 × 10^8^ cells were transduced with the library at a multiplicity of infection (MOI) of 0.3. Two days after transduction, puromycin was added (1 μg/mL) and cultured for 3 days. Then, approximately 3.0 × 10^7^ cells (422-fold coverage) were harvested as a day 0 sample. The rest of the cells were then passaged, and to maintain sufficient sgRNA coverage, the total number of cells was maintained above 3.0 × 10^7^ cells for an additional 14–21 days (eight to ten cell doublings). Genomic DNA was extracted using the Blood and Cell Culture DNA Midi Kit (Qiagen, Hilden, Germany), and a two-step PCR procedure was employed to amplify sgRNA sequences and then to incorporate deep sequencing primer sites onto sgRNA amplicons. Purified PCR products were sequenced using HiSeq 2500 (Illumina, San Diego, CA, USA). Raw and processed data files are available at the Gene Expression Omnibus database (GEO: GSE189405). The enriched or depleted genes were determined using MAGeCK-MLE (method for normalization: control sgRNAs and copy number variation) [24,25] and the results can be found in Datasets S1 (for Figure 1A) and S2 (for Figure 2A,B).

### 2.3. RNA-seq Analysis

**Figure 3:**
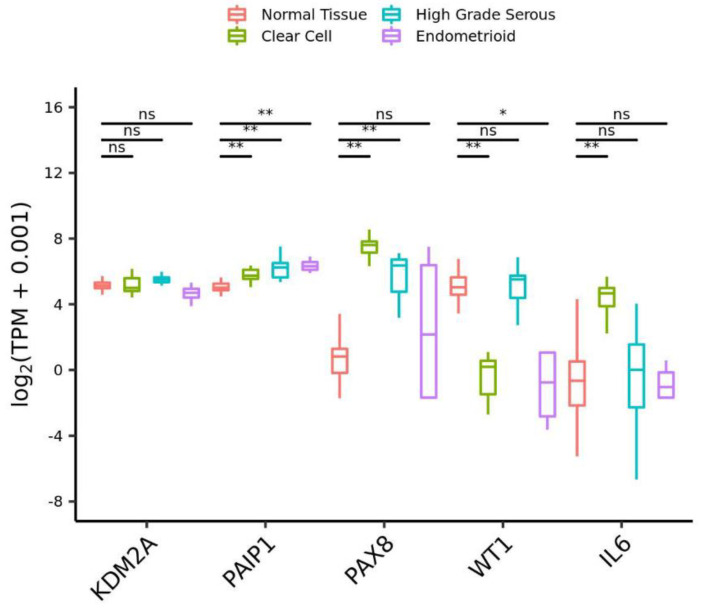
Quantification of KDM2A and PAIP1 expression from RNA-seq data. Normal tissue (from GTEx, *n* = 200) and ovarian cancer tissues prepared in our laboratory (clear cell carcinoma, *n* = 11; high-grade serous adenocarcinoma, *n* = 8; endometrioid carcinoma, *n* = 4.). PAX8, WT1 and IL6 are controls (PAX8 is known to be upregulated in ovarian cancer: PAX8 mRNA expression is 90- and 32-fold upregulated in clear cell carcinoma and high-grade serous adenocarcinoma, respectively, compared to normal tissue. PAX8 expression is ~2.7-fold upregulated in clear cell carcinoma compared to high-grade serous adenocarcinoma (*p* = 0.025, Wilcoxson test). It has also been reported that PAX8 protein expression is upregulated in several ovarian cancer cell lines [35,36]. WT1 is downregulated and IL6 is upregulated in ovarian clear cell carcinoma). TPM, transcripts per million. * *p* < 0.05, ** *p* < 0.01, ns: not significant, Wilcoxon rank sum test.

Total RNA was extracted from fresh frozen ovarian cancer tissue using TRIsure reagent (Bioline, London, UK) according to the manufacturer’s protocol and then treated with DNase I (Takara, Ohtsu, Japan) for 1 h. DNase I-treated RNA was cleaned using the NucleoSpin RNA Clean-up, Mini kit (Macherey-Nagel, Düren, Germany). RNA concentration and quality were evaluated using a NanoDrop 3000 spectrophotometer (Thermo Fisher Scientific, Waltham, MA, USA) and a Tapestation 2200 (Agilent, Palo Alto, CA, USA), respectively. All cDNA libraries were prepared using the Illumina TruSeq Stranded Total RNA and the Ribo- Zero Gold LT Sample Prep Kit according to the manufacturer’s instructions. Libraries were sequenced for 65 cycles on an Illumina HiSeq 2500 and aligned to the human reference genome build hg38 using STAR version 2.6.1d with default parameters. The gene-level expression values were estimated by RSEM version 1.3.1. For Figure 3, the ovarian normal tissue data from GTEx (Genotype tissue expression) in UCSC Xeno (https://toil-xena-hub.s3.us-east-1.amazonaws.com/download/TcgaTargetGtex_rsem_gene_tpm.gz, accessed on 1 March 2022) and our ovarian cancer tissue data were combined, and then normalized by the quantile method using the R package ‘limma’. Raw and processed data files are available at GEO (GSE189553).

### 2.4. Transfection of siRNAs

Silencer select siRNAs (PAIP1-1, s20818; PAIP1-2, s20819; KDM2A-1, s22790; KDM2A-2, s22792; Ambion, Austin, TX, USA) were transfected using Lipofectamine RNAiMAX (Life technologies, Frederick, MD, USA) 24 h after seeding. Silencer select negative control siRNA (4390843, Ambion, Austin, TX, USA) was used as a negative control.

### 2.5. Cell Titer-Glo Assay

Cell viability was determined indirectly by measuring the intracellular levels of ATP using the Cell Titer-Glo Luminescent Cell Viability Assay kit (Promega, Madison, WI, USA). Luminescence was measured using a Mithras LB 940 plate reader (Berthold Technologies, Bad Wildbad, Germany).

### 2.6. qRT-PCR Analysis

Total RNA was extracted using TRISure (Bioline, London, UK). One microgram RNA was reverse transcribed using PrimeScript RT Master Mix (Takara, Tokyo, Japan). qRT-PCR analysis of cDNA was performed on a LightCycler 480 (Roche Applied Science, Mannheim, Germany) using Syber Green PCR mastermix (Applied Biosystems, Foster City, CA, USA). The results were normalized against the values detected for GAPDH. The primers used for RT-qPCR are described in Supplementary Table S1.

### 2.7. Statistical Analysis

Statistical analyses, including two-sided t-tests and a Wilcoxon test, were performed using R version 4.0.1 (http://www.r-project.org/, accessed on 1 March 2022). A *p*-value < 0.05 was considered to be statistically significant.

## 3. Results and Discussion

### 3.1. Genome-Wide CRISPR/Cas9 Screens Using OCCC Cell Lines

To identify genes essential for the growth of OCCC, we performed CRISPR/CAS9 screens against the OCCC cell lines OVISE, TOV21G, ES2 and JHOC5 [26,27,28] using the Toronto KnockOut version 3.0 (TKOv3) library [23]. OVISE and TOV21G harbor mutations commonly observed in OCCC, including ARID1A/B and PIK3CA. ES2 has a mutation in one allele of p53 in addition to mutations in BRAF and JAK1 [29]. JHOC5 harbors a mutation in the promoter region of telomerase reverse transcriptase (TERT). We analyzed the screening results using the model-based analysis of genome-wide CRISPR-Cas9 knockout (MAGeCK) maximum-likelihood analysis (MLE) [24,25] and identified genes whose sgRNAs were significantly depleted in each cell line (Figure 1A, Figure S1 and Dataset S1). PATHWAY analysis using STRING-db revealed that the top 1000 hit genes are enriched with many genes critical for viability, including those related to mRNA processing such as spliceosome and ribosome (Figure 1B), suggesting that the screening was a success.

### 3.2. Effects of Mutations in ARID1A and PIK3CA on CRISPR/Cas9 Screening

Next, we examined whether mutations in ARID1A and PIK3CA affected gene depletion in OCCC cell lines. We found that fewer genes were depleted in ARID1A and PIK3CA mutant cells (OVISE and TOV21G, referred herein as ARID1A and PIK3CA mutant cells) than in cells without these mutations (ES2 and JHOC5, referred herein as ARID1A and PIK3CA wild-type cells) (Figure 2A). Volcano and scatter plot analysis revealed that the genes depleted specifically in ARID1A and PIK3CA mutant cells were significantly fewer than those depleted specifically in ARID1A and PIK3CA wild-type cells or those commonly depleted in both cell types (Figure 2A,B). Gene ontology (GO) analysis revealed that the genes specifically depleted in cells with ARID1A and PIK3CA mutations were enriched with those involved in the regulation of various signaling pathways, including hormone receptor, TGF-β, Notch and TOR signaling (Figure 2C). On the other hand, the genes specifically deleted in cells with wild-type ARID1A and PIK3CA were enriched with those involved in various pathways such as glutamate receptor, ephrin receptor, and B cell receptor pathways (Figure 2C). Consistent with a previous report [18], we also identified EGLN1 as a candidate gene. However, PARP1/2 and ATR, EZH2, HDAC2, HDAC6 and BRD2 were not identified in our screening (Dataset S2) [17].

### 3.3. Knockdown of Either KDM2A or PAIP1 Suppresses the Growth of OCCC Cells

Among the genes depleted in ARID1A and PIK3CA mutant cells, we selected lysine demethylase 2A (KDM2A) and poly(A)-binding protein-interacting protein 1 (PAIP1) to examine their roles in the proliferation of OCCC cell lines. According to the Cancer Cell Line Encyclopedia (CCLE) [20], KDM2A and PAIP1 are mutated in 4 and 1 cell lines out of 74 ovarian cancer cell lines, respectively. However, the significance of these mutations is unknown. KDM2A specifically demethylates lysine 36 of histone H3 and is involved in chromosome remodeling and gene transcription, and thereby regulates diverse processes such as cell proliferation, differentiation and metabolism [30,31]. STRING-db analysis of protein–protein networks revealed that KDM2A is connected to the products of transcriptional regulatory genes that are depleted specifically in ARID1A and PIK3CA mutant cells (Figure 2D). KDM2A has been reported to play important roles in the proliferation, migration and invasion of EOC cells by regulating the PI3K/AKT/mTOR signaling pathway and epithelial–mesenchymal transition (EMT) [31]. It has also been reported that KDM2A is critical for tumors such as lung cancer, breast cancer and colon cancer [30]. PAIP1 interacts with poly(A)-binding protein and the cap-binding complex eIF4A, and is involved in translational initiation and protein biosynthesis [32,33,34]. It has been reported that upregulation of PAIP1 is associated with the development and progression of tumors such as lung adenocarcinoma, pancreatic cancer and cervical cancer. We observed that PAIP1 was upregulated in OCCC compared to normal tissue (Figure 3). In contrast, KDM2A was not upregulated compared to normal tissue (Figure 3). 

**Figure 4:**
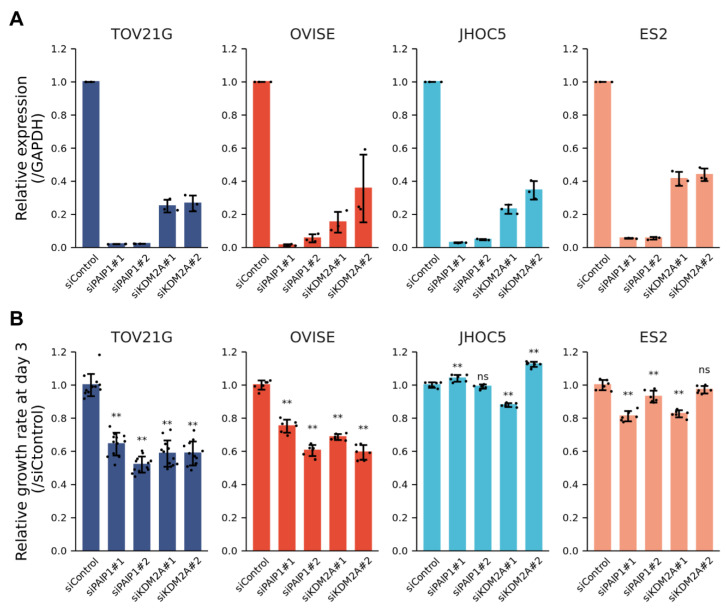
Effects of KDM2A or PAIP1 knockdown on OCCC cell proliferation. (**A**) Expression of KDM2A and PAIP1 in OCCC cell lines transfected with control siRNA or siRNA targeting KDM2A or PAIP1. Results are expressed as the mean ± s.d. (*n* = 3). * *p* < 0.05, unpaired *t*-test. (**B**) Growth of OCCC cell lines transfected with control siRNA or siRNA targeting KDM2A or PAIP1. Results are expressed as the mean ± s.d. (TOV21G: *n* = 12, OVIISE, JHOC5 and ES2: *n* = 6). ** *p* < 0.01, ns: not significant, unpaired *t*-test.

Similarly, the transcription factors connected to KDM2A (Figure 2D) were not significantly upregulated, with the exception of PAX8 (Figure S2). However, it has also been reported that KDM2A is highly upregulated in EOC tissues, and its high expression is associated with poor survival in EOC patients [31]. Furthermore, we found that siRNA knockdown of either KDM2A or PAIP1 resulted in a decrease in the proliferation of OVISE and TOV21G cells, but not ES2 and JHOC5 (Figure 4A,B). These results confirm the results of CRISPR/Cas9 screens, which suggest that KDM2A and PAIP1 may play important roles in the proliferation of ARID1A and PIK3CA mutant cells.

In the present study, we performed CRISPR/Cas9 screens against OCCC cell lines and identified candidate genes important for their proliferation. Of particular interest is the fact that different candidate genes were identified between ARID1A and PIK3CA mutant and wild-type cell lines. Thus, our results may provide a basis for new insights into the molecular mechanisms of ARID1A- and PIK3CA-mutation-induced OCCC tumorigenesis. Furthermore, we showed that KDM2A and PAIP1 may play important roles in the growth of ARID1A and PIK3CA mutant OCCC cells. However, we would need to confirm our results with a number of cell lines in future studies. In addition, we could confirm our results using JHOC5 and ES2 cells in which ARID1A and/or PIK3CA are mutated. The results of our CRISPR/Cas9 screening may be useful for the development of drugs that target OCCC.

We also analyzed data from the Cancer Dependency Map (Depmap) (https://depmap.org, accessed on 1 March 2022). Depmap data suggest that KDM2A may be important for the proliferation of most cancer cell lines, but is particularly important for ovarian cancer cell lines with mutations in ARID1A and PIK3CA. PAIP1 is suggested to contribute moderately to the growth of most cancer cell lines. Differences between Depmap and our results may be due to differences in the libraries used and statistical analysis methods.

KDM2A has a family member, KDM2B, which is very similar in structure [37]. Both have been reported to contribute to cancer cell proliferation, differentiation and senescence via H3K36 demethylation, suggesting that KDM2A and KDM2B have functional redundancy [38]. Interestingly, however, our CRISPR/Cas9 screen did not identify KDM2B as a gene essential for the growth of OCCC cell lines with ARID1A and PIK3CA mutations (Dataset S1). No family exists for PAIP1.

It has been reported that KDM2A contributes to the stemness and tumorigenicity of breast cancer cells via regulation of JAG1 and SOX2 expression [39]. In addition, knockout of KDM2A using the CRISPR/Cas9 system suppresses the growth of lung cancer cells [40]. PAIP1 is upregulated in cervical cancer and gallbladder carcinoma, and knockdown of PAIP1 decreases the tumorigenicity of these tumors [34,41]. These findings suggest that KDM2A and PAIP1 may be promising therapeutic targets in a variety of cancers other than ovarian cancer.

## Data Availability

The data that support the findings of this study are available from the corresponding author. The sequence data presented in this study are openly available in Gene Expression Omnibus database (GEO), reference number [GSE189405, GSE189553].

## Supplementary Materials

The following are available online at https://www.mdpi.com/article/10.3390/cimb44040108/s1: Figure S1: Total sgRNA counts and sgRNA mapping ratio in each sample; Figure S2: Quantification of transcription factor expression connected to KDM2A (Figure 2D); Table S1: Primers used for RT-qPCR; Dataset S1: A dataset containing the results obtained in each cell line analyzed by MAGeCK-MLE; Dataset S2: A dataset containing the results obtained in PIK3CA, ARID1A mutant cell lines (OVISE, TOV21G) and wild-type cell lines (ES2, JHOC5) analyzed by MAGeCK-MLE.
