# Supplementary material for: CRISPR/Cas9 Screening for Identification of Genes Required for the Growth of Ovarian Clear Cell Carcinoma Cells
Source: Curr Issues Mol Biol. 2022 Apr 7;44(4):1587–96. doi: 10.3390/cimb44040108 (PMC9164056; doi:10.3390/cimb44040108)
Supplement: Supplementary file 1 [file cimb-44-00108-s001.zip › Kawabata_Table_S1.pdf]

Supplementary Table S1 List of RT-qPCR primers used in this study

Name	Forward	Reverse
GAPDH	GCACCGTCAAGGCTGAGAAC	TGGTGAAGACGCCAGTGGA
PAIP1	CGGGGCTGGTGATCCATACTT	TCGCTTACGCTCTGATTCCAAAC
KDM2A	AAGAGCAGAATCTCCGTGTTGC	AGAAACCACTCCAGGGCTGC
